# Endovascular Coil Embolization of Superior Mesenteric Artery Branch Pseudoaneurysm

**DOI:** 10.7759/cureus.18014

**Published:** 2021-09-16

**Authors:** Shubra Kochar, Yiannis Sharparis, Bibek Banerjee, Maciej Karasek, Sophie Brennan

**Affiliations:** 1 Vascular Surgery, South Tyneside and Sunderland NHS Foundation Trust, Sunderland, GBR; 2 Radiology, South Tyneside and Sunderland NHS Foundation Trust, Sunderland, GBR; 3 Vascular Surgery, Newcastle University, Newcastle, GBR

**Keywords:** endovascular embolization, superior mesenteric artery, visceral arterial pseudoaneurysm, infective endocarditis, apixaban, abdominal ct angiography

## Abstract

Visceral artery pseudoaneurysms are rare but potentially life-threatening. Among visceral artery pseudoaneurysm (VAPA), the superior mesenteric artery (SMA) pseudoaneurysm is the rarest type. VAPAs are usually related with infection, inflammatory disease, trauma, or arises as a postoperative complication. Early diagnosis and endovascular or surgical intervention are key in lowering the risk of intestinal infarction and death due to potentially fatal hemorrhage. A 49-year-old man presented with cough, shortness of breath, weight loss, and an incidental finding of spontaneous SMA pseudoaneurysm with localized bleed on CT angiography. An urgent endovascular embolization was performed of the pseudoaneurysm, with an adequate outcome and recovery. SMA pseudoaneurysms associated with infective endocarditis are rare but carry a high risk of rupture and related morbidity and mortality. Direct oral anticoagulants like apixaban (Eliquis) have also been reported to cause pseudoaneurysm formation by slow and constant bleeding, which may have contributed here as a cause of pseudoaneurysm along with infective endocarditis, which was diagnosed later after endovascular embolization. The treatment can be either an endovascular, endoscopic, or open surgical approach.

## Introduction

Splenic artery visceral artery pseudoaneurysms (VAPAs) are the most frequently reported ones, and superior mesenteric artery (SMA) pseudoaneurysms are the most unlikely reported VAPAs that could be asymptomatic or present with symptoms like abdominal pain, nausea, and vomiting and local pressure symptoms, gastrointestinal hemorrhage [1-3]. Half of the patients will have a pulsatile mass or a bruit [1, 4]. Conventionally, they are associated with an inflammatory disease like pancreatitis, infection, or arise as post-surgical complication and can also occur as a known complication of traumatic damage to the artery, caused by a full-thickness slit in the arterial wall [2-4]. The condition is of clinical significance as the risk of life-threatening bleeding, acute thrombosis, and embolization of the small bowel is excessive and carries a significant mortality rate of at least 35% [5-6]. Therefore, awareness of multifactorial causes and immediate treatment is pivotal for all VAPAs, whether symptomatic, hemorrhagic or found incidentally [5]. 

## Case presentation

A 49-year-old man with British ethnicity was referred to a respiratory physician with cough, shortness of breath, and weight loss in February 2021. His CT chest showed mild centrilobular emphysema with scattered lung cysts bi basal dependent changes but no evidence of malignancy. Subsequently, CT abdomen and pelvis demonstrated an incidental finding of a 37 mm x 50 mm x 48 mm right iliac fossa well-defined mass highly suggestive of right SMA branch pseudoaneurysm with surrounding localized bleed showing contrast enhancement on arterial phase.

**Figure 1:**
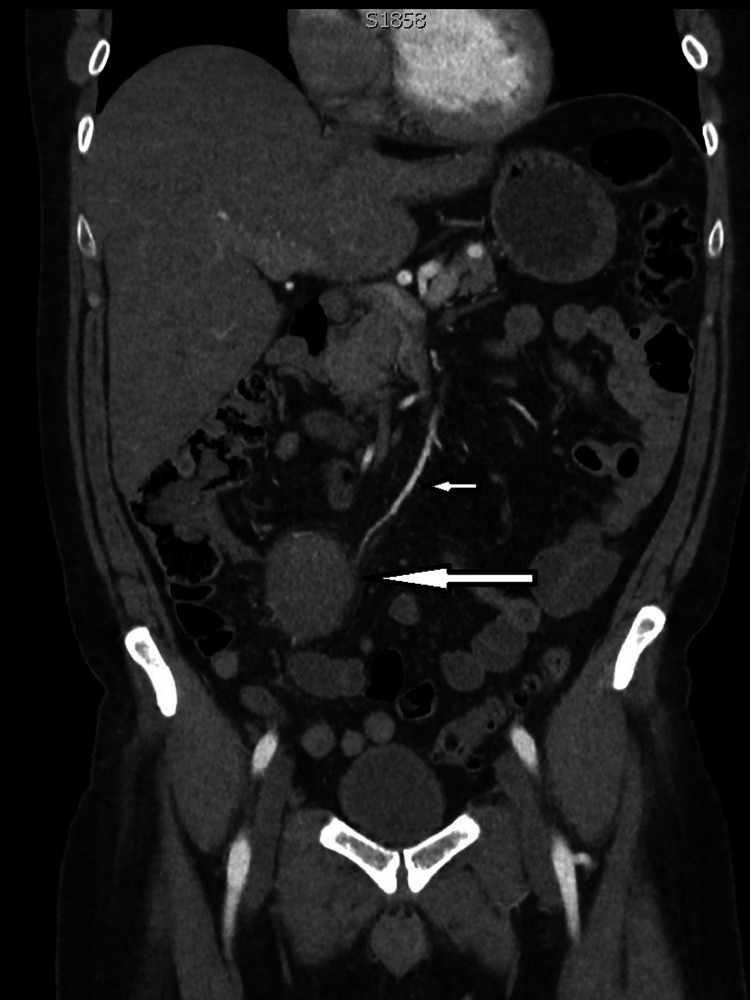
Coronal image in arterial phase of CT abdomen and pelvis which shows the feeding vessel (small arrow) into the pseudoaneurysm (big arrow) which is arising from a terminal branch of SMA. SMA, superior mesenteric artery

**Figure 2:**
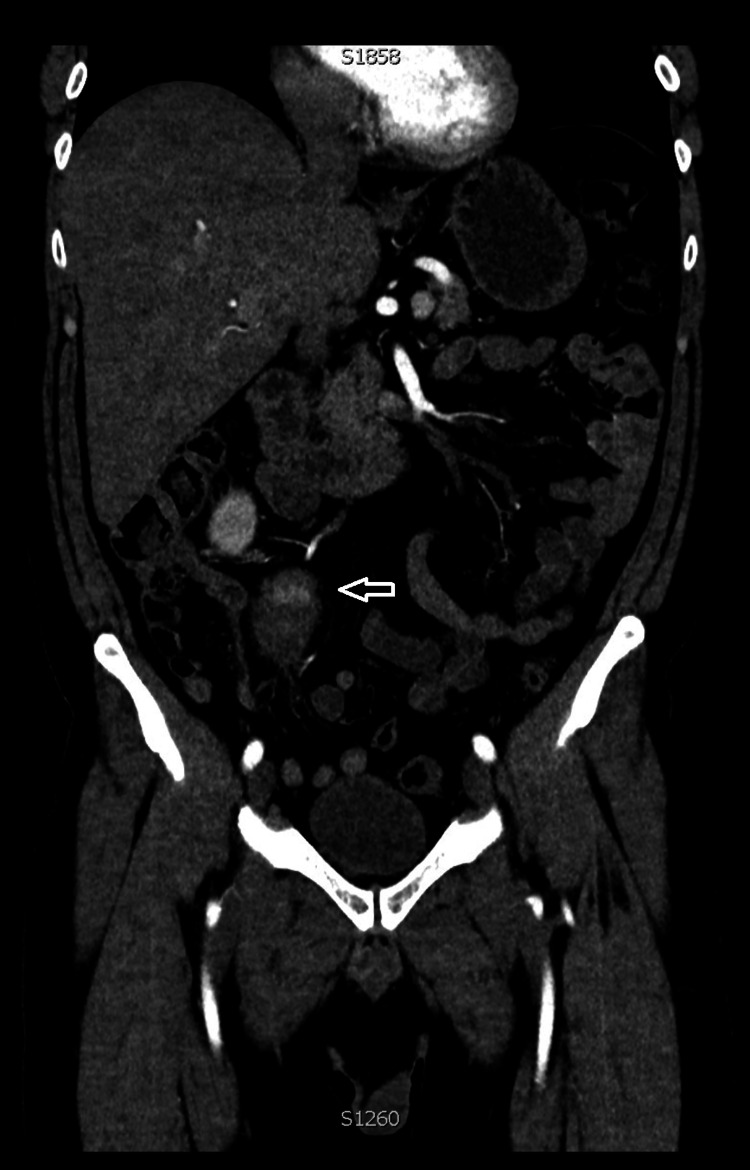
Coronal image of CT abdominal angiogram which shows the early enhancing pseudoaneurysm in the right iliac fossa (arrow).

Further patients came under the care of the Vascular team. An urgent CT abdominal angiogram taken in February 2021 showed a pseudoaneurysm arising from a terminal branch of SMA in the right iliac fossa, further suggesting an increase in size from 37 mm x 50 mm (anteroposterior [AP] × transverse [TV]) to 47 mm x 53 mm in maximum axial diameter since last CT scan and concluded as progressing SMA pseudoaneurysm (Figures 1-2).

 

**Figure 3:**
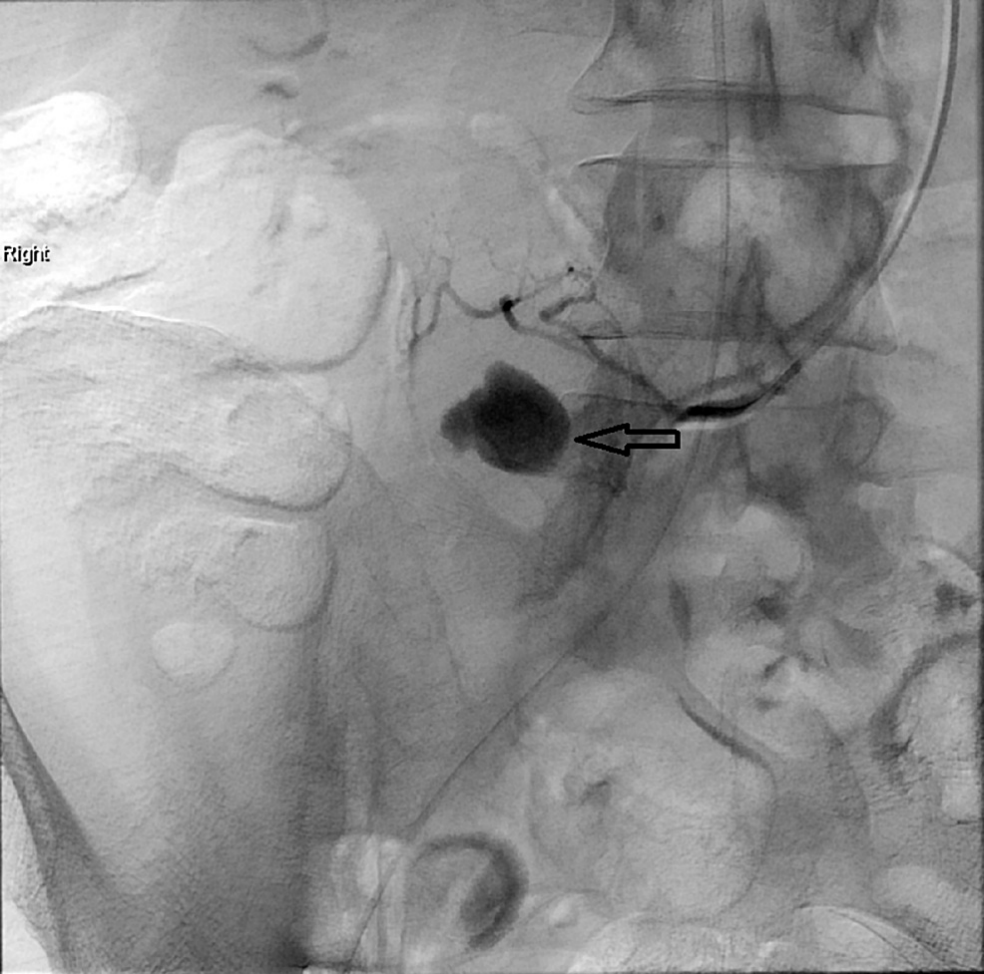
Selective SMA angiogram confirmed a right iliac fossa pseudoaneurysm supplied by the terminal branch of the SMA (arrow). SMA, superior mesenteric artery

**Figure 4:**
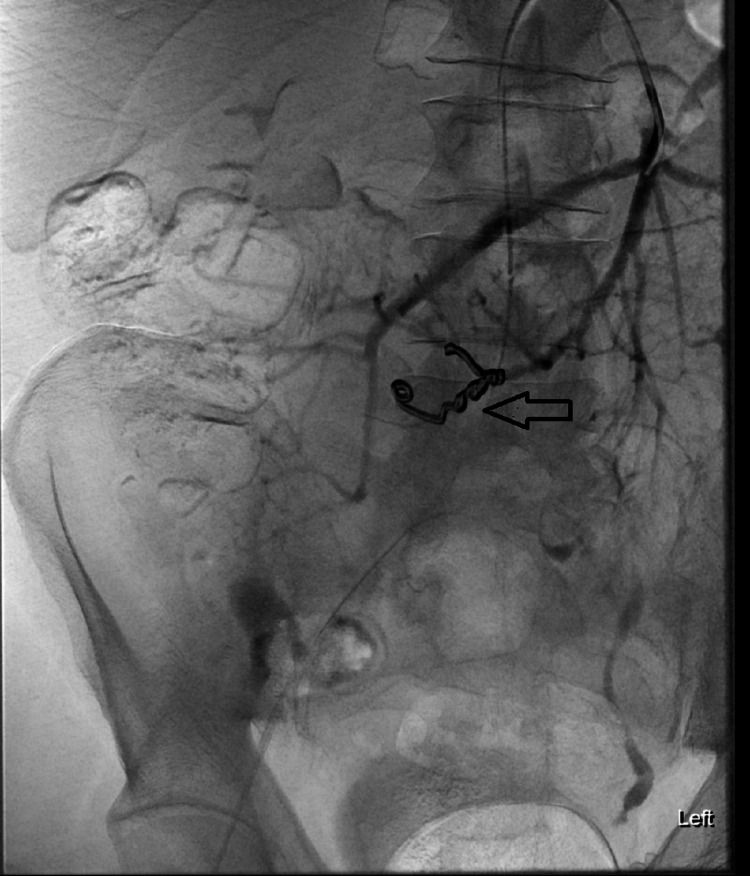
Angiogram post pseudoaneurysm coiling confirmed the coil successfully deployed and excluded flow into the pseudoaneurysm (arrow).

After discussion in the angiogram meeting in February 2021, by the team of Vascular consultants and Interventional radiologists, the decision was made in favor of endovascular embolization of pseudoaneurysm as an urgent procedure, due to the progression in size of SMA pseudoaneurysm. Supra selective embolization of the SMA pseudoaneurysm was done by the Interventional Radiology team using Right Common Femoral Artery 5F access, and MynxGrip assisted hemostasis in local anesthesia (20 mL of 0.5% levobupivacaine). The SMA and subsequently ileocolic artery were accessed using a Terumo wire and Cobra catheter. Selective SMA angiogram confirmed a right iliac fossa pseudoaneurysm supplied by the terminal branch of the SMA (Figure 3). Two fine platinum coils (Spirale) were used to embolize the pseudoaneurysm front and back door with good results and no complications (Figure 4).

 

**Figure 5:**
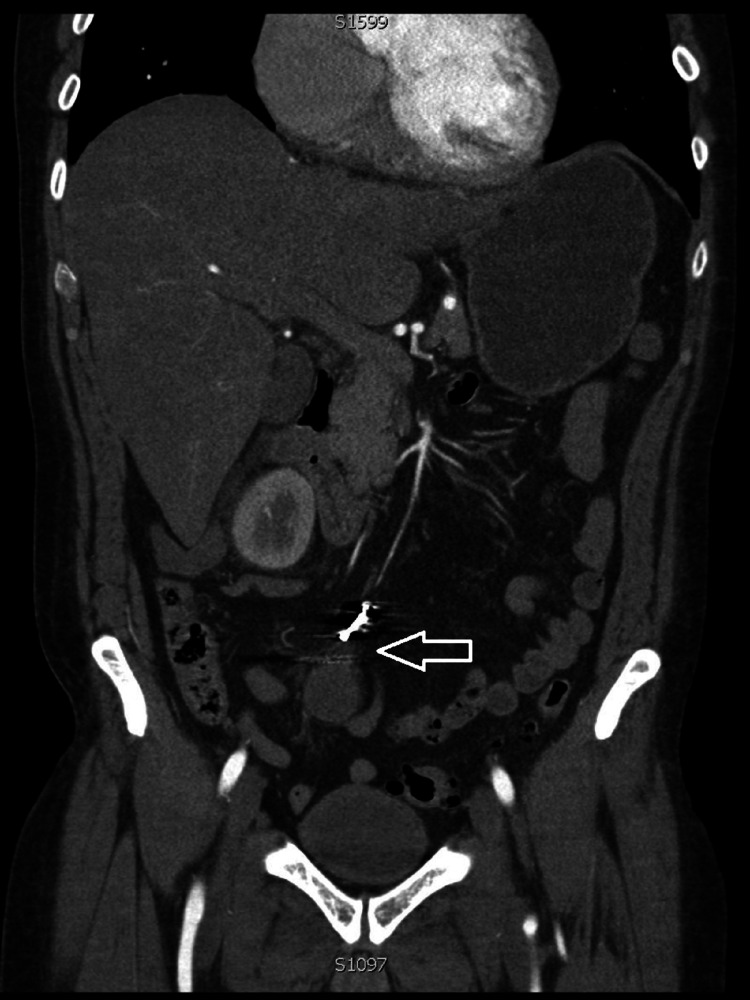
Coronal image of CT abdomen and pelvis revealed the reduced size of pseudoaneurysm with a coil in situ and no enhancement (arrow).

A post angiography run confirmed the coil successfully deployed and excluded flow into the pseudoaneurysm. His vital signs remained stable throughout. The patient tolerated the procedure and remained pain-free overall. He had cardiac comorbidity as atrial fibrillation warranting long-term anticoagulation therapy (Apixaban), and he was later diagnosed with infective endocarditis, for which he underwent valve replacement. Subsequently, apixaban is replaced with lifelong warfarin. The patient's past medical history involves type 2 diabetes mellitus, previous abnormalities found on echocardiogram, atrial fibrillation with cardioversion, diverticulitis, and significant alcohol excess [causing chronically deranged liver function tests (LFTs)]. The follow-up contrast CT abdomen and pelvis at six weeks demonstrated a reduction in the size of aneurysm sac (40 mm x 33 mm) with embolization coils in situ and no evidence of enhancement within the sac indicating successful embolization of SMA pseudoaneurysm with satisfactory visceral perfusion and no evident ischemic areas (Figure 5).

Interestingly post-embolization CT showed bilateral pleural effusion, which occurred to be caused by fluid overload resulting from infective endocarditis and heart failure. The patient was admitted again in April 2021 with Infective endocarditis with both aortic and mitral valve involvement. He was transferred to a higher cardiac center and underwent mechanical mitral valve replacement (37 mm) and aortic valve replacement (23 mm) with lifelong warfarin. He was started on IV benzylpenicillin on admission and switched to oral amoxicillin later on discharge. The ECHO report suggested moderate left ventricular systolic dysfunction with left ventricular ejection fraction as 43%-46%, dilated right ventricle with impaired function, metallic MVR/AVR in situ appears well seated, no visible aortic regurgitation, and no obvious vegetation noted with a localized rim of pericardial effusion. In this patient, the cause of visceral pseudoaneurysm appears to be infective endocarditis which was diagnosed after the intervention when the patient presented later with signs of heart failure. Surveillance for any recurrence or further aneurysmal events is arranged with a first six weekly CT scan on discharge, followed by six monthly CT angiography (CTA) for the next three to five years.

## Discussion

The SMA aneurysms and pseudoaneurysms are rarely found but can potentially lead to remarkable morbidity and mortality [4]. The SMA and branches pseudoaneurysms account for approximately 5.5% of all VAPAs, with an overall low incidence of 0.01% [3-4]. Vasculitis like polyarteritis nodosa, collagen vascular disorders like Ehlers-Danlos and Marfan's syndromes, and fibromuscular dysplasia are etiological factors associated with true SMA aneurysms, but the etiology of VAPAs is congenital, traumatic, inflammatory diseases (usually acute pancreatitis), infectious or occurred as post-surgical complications like arterial dissection [2-3]. The most common causes of SMA pseudoaneurysms are identified as trauma, and pancreatitis with infective endocarditis or uncontrolled hypertension are found as less common causes [6-7]. Life-threatening torrential hemorrhage poses a very high mortality risk and demands prompt recognition and urgent treatment. A widely used diagnostic tool for arterial dissection and aneurysms is CTA [1-2]. Several main characteristics of arteries, such as lumen (true or false) and obvious branch vessel involvement, which is crucial for diagnosis and classification, are demonstrated very well by CTA. Here in the present case, CTA remarkably demonstrated the diagnosis, provided important and beneficial information for therapy, and was lastly utilized as an important follow-up modality. Various treatment options range from open surgery to endoscopic and endovascular procedures, including endovascular coil embolization or covered stent placement [8]. Recently, there has been a transition in the management of VAPA with an increasing trend towards adopting endovascular coil embolization as a treatment option, which has shown better outcomes and reduced morbidity [9-10]. The ability to use endovascular techniques is restricted by challenging target vessel access, arterial tortuosity, and the prerequisite to conserve side branches at or near the repair site; therefore, totally endovascular repairs have been uncommonly reported [7-9]. Although scarce, apixaban, a direct oral anticoagulant, usually functions by directly inhibiting both free and clot bound factor Xa, and also prevents thrombin generation and further clotting has been reported to contribute towards spontaneous pseudoaneurysm formation with a mechanism of continuous slow-bleed [8-11]. 

## Conclusions

Visceral pseudoaneurysms are exceptionally rare, and this is one of the few reported cases of visceral pseudoaneurysms associated with infective endocarditis and/or with a direct oral anticoagulant agent like apixaban. It is necessary to manage any VAPAs with top priority as it carries a very high risk of rupture and potentially serious hemorrhage. A total endovascular repair was considered, and endovascular coil embolization was done. Endovascular coil embolization is an effectual and safe technique out of all options available for absolute management in treating VAPAs, especially for the high-risk operative candidates.
